# Tnni3k Is Cardioprotective in Viral Myocarditis

**DOI:** 10.3390/jcdd13020069

**Published:** 2026-01-30

**Authors:** Kelsey Tjen, Ruolan Song, Baylee C. Westbury, Lunndon A. Lewis, Ge Tao, Kristine Y. DeLeon-Pennell, Katelyn A. Bruno, Henry M. Sucov

**Affiliations:** 1 Department of Regenerative Medicine and Cell Biology, Medical University of South Carolina, Charleston, SC 29425, USA; tjen@musc.edu (K.T.); ruolan.song@emory.edu (R.S.); westburb@musc.edu (B.C.W.); lewislu@musc.edu (L.A.L.); taog@musc.edu (G.T.); 2 Division of Cardiology, Department of Medicine, Medical University of South Carolina, Charleston, SC 29425, USA; deleonky@musc.edu; 3 Division of Cardiovascular Medicine, Department of Medicine, University of Florida, Gainesville, FL 32608, USA; katelyn.bruno@medicine.ufl.edu

**Keywords:** viral myocarditis, CVB3, Tnni3k

## Abstract

The severity of viral myocarditis in humans and mice is variable between individuals. Numerous observations demonstrate the influence of host genetics on disease course, but few genes have actually been identified to have such properties. In past work, mouse strains that are sensitive or resistant to severe disease were used to map the viral myocarditis susceptibility locus *Vms1*. Here, we demonstrate that *Tnni3k*, one of the genes within the *Vms1* locus, influences the severity of disease following inoculation with coxsackievirus CVB3. Compared to disease-resistant C57BL/6J wild-type mice, strain-matched *Tnni3k* knockout mice showed higher cardiac inflammation and, in particular, a greater infiltration of macrophages into the heart. Long-term damage associated with viral infection was comparable in mice of both genotypes. Use of a second mouse line engineered with a point mutation to encode a kinase-dead version of Tnni3k showed the same elevated inflammation as the full null. These results identify Tnni3k and its kinase activity as being protective in modulating the acute phase of inflammatory response to CVB3 infection in the heart.

## 1. Introduction

Myocarditis, or inflammation of the myocardium, is a leading cause of heart failure and sudden cardiac death. The incidence of myocarditis is estimated to be approximately 1.5 million cases worldwide per year, with an unknown (and likely much larger) number of undiagnosed and/or subacute cases. Of the several circumstances that can lead to myocarditis, viral infection is the most common etiology. The viruses most often implicated include adenoviruses and enteroviruses, both of which are highly prevalent among the general population [1,2,3], but many others elicit a similar pathology [4,5]. Coxsackievirus B3 (CVB3), the most common enterovirus causing human myocarditis, is often used to induce myocarditis in murine studies.

Viral myocarditis begins with initial host infection via any of several routes, followed by virus migration to the heart and infection of cardiomyocytes (CMs). Replication of the virus induces cardiac damage, which initiates the successive recruitment of immune cells into the myocardium. Most patients clear the infection with no long-term consequences. However, approximately 10% of patients develop a chronic myocarditis [6], which at the tissue level can involve cardiac remodeling and may further progress to the development of dilated cardiomyopathy and heart failure and/or arrhythmia and sudden cardiac death [7,8]. The features that determine variable susceptibility and progression to severe disease are mostly unknown, but clinical observations imply the relevance of patient genetic variation to variable human disease outcome [9,10,11].

Because of their experimental accessibility and consistency, mouse studies make it practical to rigorously explore the genetics and mechanistic features that underlie viral myocarditis. Different inbred mouse strains are polymorphic in a vast number of genes and thus embody a broad range of natural (as opposed to engineered) genetic variation. Inbred mouse strains differ significantly in response or outcome to CVB3 infection [12,13,14,15], clearly implicating (as in humans) a role for host genetics in myocarditis susceptibility. C57BL/6J mice are resistant to viral myocarditis and A/J mice are susceptible [13]. A 2007 mapping study in second-generation (F2) intercrossed mice aimed to identify novel genetic factors associated with myocarditis that differ between the inbred strains C57BL/10J (closely related to C57BL/6J) and A/J [16]. This study identified a large (approximately 30 Mb) locus on mouse chromosome 3, named *Vms1* (viral myocarditis susceptibility 1), linked to severe myocardial lymphocyte infiltration and sarcolemmal damage. This locus was further refined in a 2011 study to a 9 Mb range, referred to as *Vms1.1* [14], and is recessive in A/J mice.

*Tnni3k*, one of the genes within the *Vms1.1* locus, is a mixed serine/threonine and tyrosine kinase with cardiomyocyte-specific expression [17]. It is the only cardiomyocyte-specific kinase, and is highly conserved across all vertebrates. Nonetheless, the *Tnni3k* gene is not required for heart function or viability, as evidenced by the following observations. Many inbred mouse strains are homozygous for a natural loss-of-function polymorphism [18], yet they appear normal and healthy with no prominent baseline cardiac abnormalities. An engineered null mutation of the *Tnni3k* gene created in C57BL/6J mice was also viable with little perturbation of cardiac (or other) biology [19]. Homozygous loss-of-function alleles of the human *TNNI3K* gene have been identified, including a fairly prevalent I686T polymorphism that is severely hypomorphic in kinase activity [20], without reported phenotypic consequence, and the canine homolog is naturally mutated to a fully kinase-dead allele in some individuals of the West Highland White Terrier breed [21], also without known consequence.

The Tnni3k protein is modular, with an N-terminal ankyrin repeat domain, a central kinase domain, and a C-terminal unstructured domain. Because ankyrin repeat domains can serve as a protein–protein interface, it was unknown if the protein requires its kinase activity (this was not unreasonable, the kinase activity of the fairly closely related kinase ILK has been shown to be dispensable [22]). Most protein kinases have a lysine residue at a conserved location that coordinates the terminal phosphate of ATP as it is transferred to a substrate, and mutation of this lysine to arginine in other kinases abolishes kinase activity. In mouse Tnni3k, this lysine is at position 489 (human position 490), and we and others have shown in an in vitro assay that a K489R mutation abolishes Tnni3k kinase activity. We created a K489R allele in C57BL/6J mice and showed that this mutation phenocopies the effects of the full null allele in CM polyploidy [23] and in CM aspect ratio [20]. Its requirement for kinase activity in other processes has not been assessed.

A/J mice (susceptible to viral myocarditis) are homozygous for the natural *Tnni3k* null allele, whereas resistant C57BL/6J (and C57BL/10J) mice express the functional gene. This is consistent with the recessive nature of the *Vms1* allele in A/J mice, as defined in the genetic mapping analysis described above. In mouse intercross studies, the large number of polymorphic genes that differ between two inbred strains is an advantage for mapping but a disadvantage for conducting a definitive test of the role of any candidate gene in a process of interest. Here in this study, we investigated the involvement of Tnni3k in viral myocarditis. We used an engineered *Tnni3k* gene knockout line that was created and maintained on a C57BL/6J- background [19]. By comparing to wild-type C57BL/6J mice (i.e., strain background matched), we demonstrate a cardioprotective role for Tnni3k in viral myocarditis. We further show that Tnni3k kinase activity is critical for this response.

## 2. Materials and Methods

Animals. All animal protocols and procedures complied with NIH guidelines and were approved by the Institutional Animal Care and Use Committee (IACUC) of the Medical University of South Carolina (#2018-00642). *Tnni3k* KO and K489R alleles used in this study were previously described [23,24]. All Tnni3k alleles in this study originated and were maintained on an inbred C57BL/6J-background with WT mice (JAX #000664) obtained from The Jackson Laboratory (Bar Harbor, ME, USA). Specifically, the original conditional *Tnni3k* allele made on a C57BL/6 background [19] was imported to our colony in 2014 and crossed to a *Sox2Cre* line (inbred on a C57BL/6 background; JAX #008454) to create a full null (KO) allele [24]. KO mice and K489R mice were typically maintained as homozygous lines for several generations by intercrossing, but were periodically outcrossed to wild-type C57BL/6J mice obtained from JAX to prevent genetic drift. Wild-type (WT) control mice used in this study were bred in our colony in a similar manner.

CVB3-Induced Myocarditis Model. Coxsackievirus B3 (CVB3; Nancy strain) was originally obtained from the American Type Culture Collection (ATCC) (Manassas, VA, USA) and grown in Vero cells, as previously described [25]. Male mice between 7 and 9 weeks old were inoculated intraperitoneally with 10^3^ plaque, forming units of heart-passaged virus stock prepared as described [26].

Histology. Freshly isolated mouse hearts were rinsed in ice-cold Kruftbrühe (KB) buffer (70 mM potassium aspartate, 40 mM KCl, 15 mM KH_2_PO_4_, 10 mM glucose, 10 mM taurine, 0.5 mM EGTA, 10 mM sodium pyruvate, 10 mM HEPES, 5 mM BDM, 0.5% BSA, pH 7.6) to arrest in diastole, manually cut in half longitudinally to obtain a 4-chamber view, pre-fixed in 4% formaldehyde in KB buffer for 30 min at room temperature, and fixed in 10% phosphate-buffered formalin for 48 h at room temperature. Hearts were dehydrated through increasing ethanol concentrations and embedded in paraffin for histological analysis. Five micron sections were obtained on a HistoCore Autocut (Leica, Wetzlar, Germany). Sections were stained with hematoxylin and eosin (H&E) to detect immune cell infiltration or picrosirius red to detect collagen and images captured with a Leica MC170 HD camera (Leica, Wetzlar, Germany). To quantify inflammation and fibrosis, ilastik (version 1.4.0) [27], a machine learning image analysis software, was used to create a mask over clusters of hematoxylin-stained nuclei, representing areas of immune cell infiltration, or over streaks of picrosirius red-stained collagen fibers. The area of this mask was quantified using ImageJ (version 2.14.0) [28] and divided by total area of ventricular tissue. Each data point is the average of 2–3 sections, at least 200 microns apart, from one heart sample.

Immunohistochemistry. Antigen retrieval was performed in a microwave oven for 25 min in a pressure cooker using citrate antigen retrieval buffer (9 mM sodium citrate, pH 6.0). After blocking with 1% blotting grade milk (Fisher Scientific, sc-2325, Waltham, MA, USA), heart tissue sections were incubated at 4 °C overnight in a humid chamber with primary antibody: rat anti-mouse Mac-3 (1:600, Cedarlane Labs, CL8943AP, Burlington, ON, Canada). The following day, sections were incubated at room temperature for 2 h in a humid chamber with secondary antibody: goat anti-rat HRP (1:200, Jackson ImmunoResearch, 112-035-003, West Grove, PA, USA). Extent of staining in histology sections was quantified using ilastik, as described above.

Flow Cytometry. Freshly isolated mouse hearts were rinsed in ice-cold KB buffer to arrest in diastole and incubated in type II collagenase (100 mg/heart, Gibco, 17101015, Thermo Fisher Scientific, Waltham, MA, USA) in 1x PBS at 37 °C, with mechanical dissociation every 10 min, until there was no undissolved tissue (approximately 1 h). Cells were filtered through a 40 µm cell strainer (VWR 732-2757) and red blood cells lysed with ACK lysis buffer (154.4 mM ammonium chloride, 10 mM potassium bicarbonate, 97.3 µM EDTA, pH 7.2). Cells were resuspended in PEB buffer (1x PBS, 2 mM EDTA, 0.5% BSA, pH 7.2) and incubated with FcR blocking reagent (Miltenyi, 130-092-575, Bergisch Gladbach, Germany) for 20 min at 4 °C to block nonspecific binding. Cells were stained with Viobility Fixable Live/Dead Dye (1:100, Miltenyi, 130-130-404, Bergisch Gladbach, Germany) to exclude dead cells and debris. To prepare single-channel controls for flow cytometry compensation, isolated splenocytes were mixed with cardiac preparations in a 1:1 ratio and stained in parallel. Cells were stained with surface-marker antibodies for 20 min at 4 °C protected from light: RB780 rat anti-mouse CD45 (1:100, BD Biosciences, 569150, Franklin Lakes, NJ, USA), FITC rat anti-mouse Ly6G (1:25, BD Biosciences, 551460, Franklin Lakes, NJ, USA), PerCP-Cyanine5.5 rat anti-mouse Ly6C (1:100, eBioscience, 45-5932-82, Waltham, MA, USA), and PE hamster anti-mouse CD3e (1:25, eBioscience, 12-0031-82, Waltham, MA, USA). To prevent spread of potentially virulent CVB3, cells were fixed in 2% paraformaldehyde (PFA) in 1x PBS for 20 min at room temperature. Flow cytometry was performed using a MACSQuant Analyzer 10 Flow Cytometer (Miltenyi, Bergisch Gladbach, Germany) and analyses were performed with MACSQuantify software (Miltenyi, version 3.0.2). Debris was excluded based on the SSC-A vs. FSC-A plot, doublets removed by FSC-H vs. FSC-A, dead cells excluded with live/dead dye staining, and immune cells gated based on SSC-A and their respective fluorescent signals. Gates were confirmed using fluorescent minus one (FMO) samples and backgating to the SSC-A vs. FSC-A plot.

Quantitative Reverse Transcriptase PCR. At harvest, hearts were cut in half longitudinally. One half was flash frozen in liquid nitrogen and stored at −80 °C for RNA isolation. Hearts were mechanically homogenized and RNA extracted by TRIzol (Invitrogen, 15596026, Waltham, MA, USA). RNA was converted to cDNA using random hexamers and SuperScript III Reverse Transcriptase (Invitrogen, 18080044, Waltham, MA, USA and assessed using primers and SsoAdvanced™ Universal SYBR^®^ Green Supermix (Bio-Rad, 1725271, Hercules, CA, USA) on a Bio-Rad CFX96 Connect/Opus real-time quantitative PCR machine (Hercules, CA, USA). Data are shown as relative gene expression (RGE) normalized to the reference gene hypoxanthine phosphoribosyltransferase 1 (*Hprt*). All primers were purchased from ThermoFisher (Waltham, MA, USA). Primers were designed to span exon–exon junctions and primer pairs were required to be separated by at least one intron on the corresponding genomic DNA. Gene expression was analyzed by assessing comparative quantification, which utilizes cycle threshold (C_t_) for each primer to calculate the delta C_t_ (ΔC_t_), which is the threshold cycle comparison between the gene of interest and the reference gene. A second ΔC_t_ normalizes to a control; in this study, uninfected animals were used as control. These were then used to calculate the relative gene expression using the formula $RGE=\;2^{-\Delta \Delta C_{t}}$.

Primer Sequences. CVB3 Forward 5′-CCCTGAATGCGGCTAATCC-3′; CVB3 Reverse 5′-ATTGTCACCATAAGCAGCCA-3′; *Hprt* Forward 5′-TCCCAGCGTCGTGATTAGC-3′; *Hprt* Reverse 5′-TGATGGCCTCCCATCTCCTT-3′.

Statistical Analysis. Data comparing two groups were analyzed using a two-tailed Student’s *t*-test, performed in Excel. Data are expressed as mean +/− standard deviation.

## 3. Results

In this study, we used heart-passaged CVB3 (Nancy strain) to induce viral myocarditis; this preparation initiates a disease course in mice that resembles that in humans [29]. Due to the known influence of age [30] and sex hormones [30,31,32,33,34,35] on susceptibility to myocarditis, all experiments were conducted on male mice infected between the ages of 7–9 weeks.

### 3.1. Tnni3k Is Cardioprotective in Viral Myocarditis

**Figure 1:**
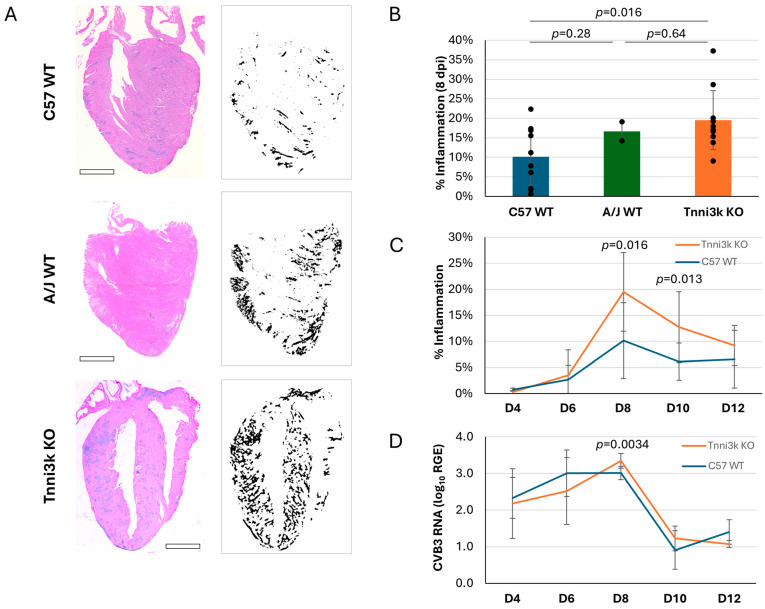
*Tnni3k* KO mice are susceptible to more severe myocarditis. (**A**). Representative H&E images of C57BL/6J WT, A/J WT, and *Tnni3k* KO hearts 8 dpi (left) and their corresponding ilastik inflammation “masks” (right). Scale bars: 1 mm. (**B**). Percent inflammation of C57BL/6J wild-type (C57 WT, n = 10), A/J wild-type (A/J WT, n = 2), and *Tnni3k* knockout (Tnni3k KO, n = 10) animals 8 dpi. (**C**). Myocarditis is defined as area of immune cell infiltration divided by total area of ventricular tissue. Percent inflammation at 4, 6, 8, 10, and 12 dpi (C57 WT n = 6, 8, 10, 12, 3; *Tnni3k* KO n = 6, 9, 10, 9, 3). The same day 8 data shown in B for C57 WT and *Tnni3k* KO are incorporated into this panel. (**D**). Relative gene expression (log_10_) of viral protein gene VP1 at 4, 6, 8, 10, and 12 dpi.

As myocarditis is defined by immune cell infiltration of the heart, we first looked at infiltration in infected mice by H&E staining, in which immune cell clusters are recognizable by their high density of hematoxylin-stained nuclei (Figure 1A). Using immune cell infiltration as an index, we generated an inflammation score for every stained section by dividing the area of immune cell infiltration by the total area of ventricular tissue. Each mouse data point is the average of 2–3 non-nearby sections scored in this manner; section-to-section variation was low (Supplementary Figure S1), indicating that immune cells, although clustered, were distributed relatively uniformly across the ventricle. Confirming past studies, A/J mice demonstrated greater inflammation (16.65% vs. 10.16%) (Figure 1B) within the heart at 8 dpi (days post-infection). Importantly, *Tnni3k* KO mice showed a similar heightened susceptibility (19.50%) to inflammation. Because the knockout mice are strain background-controlled to the wild-type mice, this is a rigorous demonstration that Tnni3k controls susceptibility to viral myocarditis. Specifically, the presence of Tnni3k was protective and its absence was detrimental. This result identifies *Tnni3k* as being at least one, if not the only, gene in the *Vms1* locus that controls this phenotype.

We evaluated the time course of immune cell infiltration over the acute phase of myocarditis (Figure 1C). As previously described in wild-type C57BL/6J mice [25], levels of inflammation were low (<5%) at days 4 and 6, peaked around day 8, before dropping to a low but still present level thereafter. The course of inflammation in *Tnni3k* KO animals mimicked that of WT animals, but to a more severe degree. At days 8 and 10, inflammation in *Tnni3k* KO mice was approximately double that of WT animals.

Tnni3k clearly influences immune cell recruitment into the infected myocardium, although there could be several mechanisms by which this could occur. To address whether Tnni3k controls viral expansion within cardiomyocytes, we measured the level of viral genomic RNA present within the heart by qPCR in samples of the same hearts used for H&E staining. As shown in Figure 1D, both genotypes demonstrated a similar profile, with early presence of virus peaking at day 8 before being cleared by day 10. There was a mild increase in *Tnni3k* KO mice relative to WT at 8 dpi, which was of statistical significance, although of uncertain biological significance, especially as the mere presence of viral RNA is not evidence of functional virus (i.e., noninfectious viral remnants persist after active virus is cleared [36]). These data demonstrate that Tnni3k does not prominently influence the course of virus infection or clearance.

### 3.2. Characterization of the Immune Cell Profile in Infected Hearts

**Figure 2:**
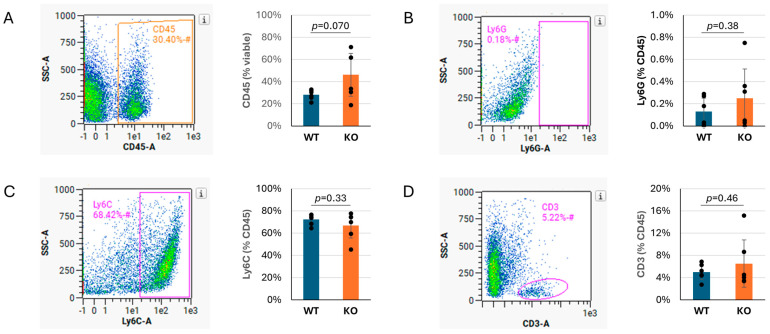
Ly6C^+^ monocytes/macrophages make up the majority of immune infiltration in both genotypes. (**A**). Density plot showing percentage of viable cells that are CD45^+^, quantified to the right. (**B**–**D**). Representative density plots showing percentage of CD45^+^ cells that are Ly6G^+^ (**B**), Ly6C^+^ (**C**), or CD3^+^ (**D**), with quantification to the right. n = 6 for each genotype. The *x* axis in flow cytometry plots is in log10 scale. Gated areas of the plots are indicated by rectangles or ovals.

To determine if Tnni3k influences the proportions of different subtypes of immune cells recruited to the infected heart, or if all immune cells were increased in general, we performed flow cytometry on WT and *Tnni3k* KO animals at 8 dpi. This time point was based on the H&E observation (Figure 1C) that showed peak immune cell infiltration at this time. The percentage of cells expressing the pan-immune cell marker CD45 was 1.6-fold elevated in Tnni3k KO hearts, consistent with the difference seen by histology, although as measured by flow cytometry this difference did not reach statistical significance (Figure 2A). Within the population of CD45^+^ immune cells, subpopulations of Ly6G^+^ neutrophils/granulocytes, Ly6C^+^ monocytes/macrophages, and CD3^+^ T cells were the same in both genotypes (Figure 2B–D). This outcome implies that Tnni3k does not act to selectively recruit a unique spectrum of immune cells (at least to the extent defined by these broad markers), but rather influences the overall level of immune cell recruitment. Approximately 25% of CD45^+^ cells in both genotypes were uncharacterized in this analysis; this likely includes eosinophils, B cells, NK cells, and others. Immune cell populations in uninfected mice of both genotypes (Supplementary Figure S2) were similar, as previously reported [37].

**Figure 3:**
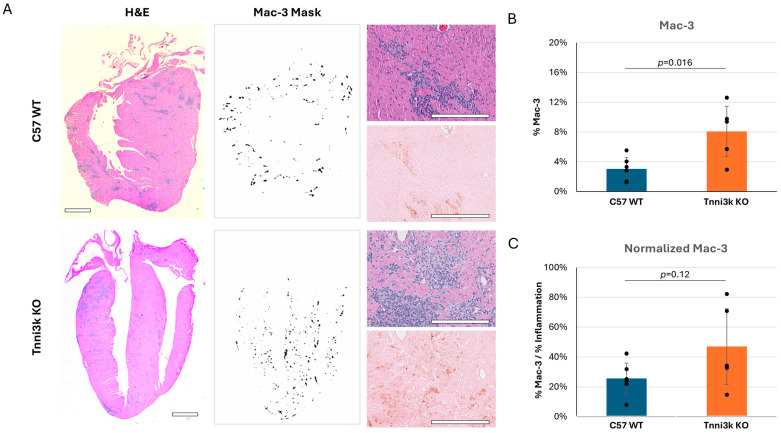
Mac-3 staining supports flow cytometry data, showing macrophages make up a prominent portion of immune infiltrates. (**A**) Representative H&E images of C57 WT and *Tnni3k* KO hearts 8 dpi (left, 2x; top right, 20x) and adjacent sections immunostained for Mac-3 (middle, 2x ilastik mask; bottom right, 20x). Scale bars: 1 mm in low-magnification views, 200 µm in high-magnification views. (**B**) Quantification of Mac-3 staining normalized to tissue area (WT n = 6, KO n = 5). (**C**) Mac-3 staining normalized to inflammation (based on ilastik masks of adjacent H&E stained sections).

Of note, Ly6C^+^ monocytes/macrophages made up the overwhelming majority (~70–75%) of the CD45^+^ immune cells in the day 8 infected hearts of both genotypes (Figure 2C), pointing to their prominent role in responding to viral infection. To support this observation, we performed immunohistochemistry by staining sections for the macrophage-specific marker Mac-3. Immunostaining confirmed an abundant number of cells expressing Mac-3, following the same pattern as observed by H&E, although clearly these clusters of immune cells are heterogeneous in composition (Figure 3). When quantified to heart tissue area, Mac-3 staining in *Tnni3k* KO mice was more than double that of WT mice (Figure 3B). When normalized to area of immune cell infiltration (as defined by H&E staining), we calculated a 1.7-fold increase in KO hearts. In principle, Mac-3^+^ area normalized to inflammation area might be comparable to the ratio of Ly6C^+^ cells to CD45^+^ cells in flow cytometry, the latter of which was equivalent in WT and KO (Figure 2C). As Mac-3 can also stain resident cardiac macrophages, the difference between Mac-3^+^ staining by histology and Ly6C^+^ cells by flow cytometry may indicate increased expansion of resident cardiac macrophages with *Tnni3k* deletion. Alternatively, because some T cells express Ly6C, there may be enhanced recruitment of a Ly6C^+^Mac-3^−^ subclass of immune cells in KO mice.

### 3.3. Tnni3k Does Not by Itself Influence the Viral Myocarditis Terminal Phenotype

**Figure 4:**
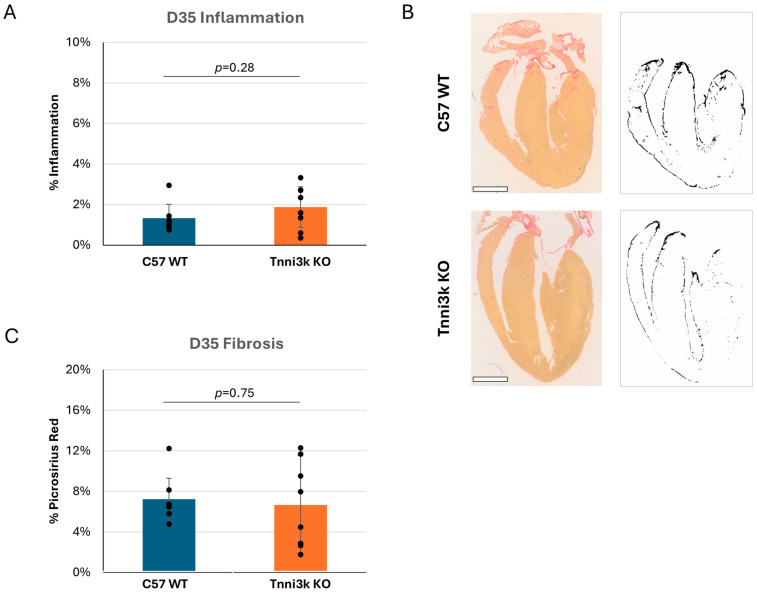
*Tnni3k* KO does not influence chronic myocarditis. (**A**) Inflammation (defined as in Figure 1) normalized to tissue area at 35 dpi is low and comparable between genotypes. (**B**) Representative images of C57 WT and *Tnni3k* KO hearts 35 dpi stained with picrosirius red for fibrosis (left) and of the ilastik masks generated for quantification (right). Scale bars: 1 mm. (**C**) Quantification of picrosirius red staining at 35 dpi (C57 WT n = 7, Tnni3k KO n = 8).

While most humans clear a viral infection of the heart without damage, a subset progress to cardiomyopathy and even failure. Fairweather et al. previously showed that some susceptible strains of mice (e.g., BALB/c, which carries the natural *Tnni3k* null allele) develop dilated cardiomyopathy by 35 dpi, while C57BL/6 mice do not [25]. With the availability of *Tnni3k* KO mice on a C57BL/6J-background, we tested to see if Tnni3k is responsible for this late viral myocarditis phenotypic outcome. H&E staining of hearts at 35 dpi showed that immune cells were largely cleared in both genotypes (Figure 4A). Although we did not perform echocardiography to evaluate function and chamber dimension, histology showed no obvious dilation or other structural heart abnormality. Fibrosis is a common response to injury, especially following recruitment of macrophages. We stained sections with picrosirius red and saw low (6–7%) but not significantly different evidence of fibrosis (Figure 4C). These observations indicate that Tnni3k plays a larger role during the acute phase of myocarditis. Strains that develop chronic heart pathology, many of which carry the natural *Tnni3k* null allele, do so for reasons beyond Tnni3k alone.

### 3.4. Tnni3k Kinase Activity Is Required for Resistance to Viral Myocarditis

**Figure 5:**
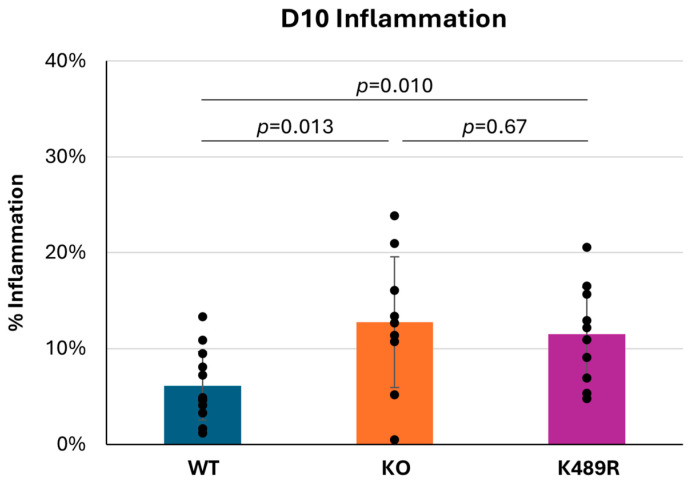
*Tnni3k* K489R kinase-dead mice and full null mice are equally susceptible to severe myocarditis. Percent inflammation at 10 dpi (C57 WT n = 12, Tnni3k KO n = 9, Tnni3k K489R n = 10). Data for WT and KO are the same as in Figure 1C.

As noted above, the fact that Tnni3k is a functional kinase does not necessitate that its kinase activity is required for its in vivo effects. We previously created a kinase-dead *Tnni3k* K489R allele in C57BL/6J-inbred mice and showed that this allele phenocopies the full null allele in some tested features [20,23]. To address the requirement for kinase activity in viral myocarditis, we infected Tnni3k kinase-dead K489R mice with CVB3. At 10 dpi, K489R mice demonstrated a level of inflammation comparable to the *Tnni3k* KO mice and higher than WT animals (Figure 5). This demonstrates that the kinase-dead animals are equally susceptible to myocarditis and that kinase activity is essential to Tnni3k’s protective role in viral myocarditis.

## 4. Discussion

The controlled experimental studies that are possible to undertake in mice allow dissection of the variable and sometimes extreme nature of disease course in human viral myocarditis. It is clear that different disease features are intensified or minimized in different inbred mouse strains, but the specific genes responsible for these outcomes are challenging to identify. This study shows that *Tnni3k*, one gene within the previously mapped *Vms1* locus, has a prominent influence on the early immune cell response to viral challenge. Our study was limited to male mice, which show a more pronounced myocarditis phenotype; we presume but have not shown a similar role in females.

The *Tnni3k* gene was possible to identify in mouse strain crosses because a natural loss-of-function allele became fixed to homozygosity in the early derivation of many common inbred mouse strains. Absence of Tnni3k has no obvious consequence in viability, reproduction, or overall health at baseline conditions, implying that the role of the gene and protein may only become manifest in response to specific physiological or environmental challenges. Here, we show such a role in mediating the inflammatory response to CVB3 viral infection. The availability of strain-matched wild-type and *Tnni3k* mutant mouse lines allowed this analysis to be conducted in a properly controlled and rigorous manner.

Viruses that cause myocarditis spread widely in the body after initial infection, yet viral myocarditis is a disease specifically of the heart. This implies cardiac-specific elements. One of the appealing features of identifying *Tnni3k* as a regulator of viral myocarditis is that this gene is expressed only in cardiomyocytes, and thus accounts for one part of the mechanism that results in the heart-specific inflammation following viral infection. To be sure, the consequences of Tnni3k deficiency in this study are limited to the severity of early immune cell infiltration but without the long-term damage that is seen in some people and in some mouse strains. C57BL/6J mice are known to be resistant to severe inflammation and to the development of chronic disease, and our study shows that absence of Tnni3k does not by itself cause C57BL/6J-background mice to develop late-stage pathology. It is likely that other allelic variants that differ between inbred mouse strains are more relevant to later disease phenotype. As noted above, many inbred mouse strains that have been shown to be susceptible to severe disease in both the acute and chronic periods naturally lack Tnni3k. If genes that are responsible for chronic pathology are identified, it will be worthwhile to assess if their manifestation in the chronic period requires the earlier elevated inflammation associated with the absence of Tnni3k.

It remains unclear how Tnni3k activity modulates the inflammatory response to viral infection in the heart. Our analysis of K489R mice shows that Tnni3k kinase activity is required, but other than itself (by autophosphorylation), no authentic Tnni3k kinase substrate has yet been confirmed. Several observations suggest its involvement in oxidative stress response [19,38], and Tnni3k augments adrenergic signaling in CMs [33], but the specific kinase substrates involved in these activities, and whether these or other mechanisms are relevant in inflammatory response, are unknown.

The composition of the infiltrating immune cells during myocarditis is of great interest. Macrophages make up the majority of infiltrating immune cells at day 8 post-infection (Figure 2C). Many studies have examined the balance between pro-inflammatory mediators clearing a viral infection and anti-inflammatory cells restricting cardiac damage. Greater expression of pro-inflammatory markers and a traditionally M1 macrophage polarization was associated with more severe cardiac inflammation after CVB3 infection [34], while transfer of M2 macrophages alleviated myocardial disease [35]. In this study, Ly6G^+^ granulocytes and neutrophils were predictably rare at day 8 (Figure 2B), but these play important roles in the activation and recruitment of macrophages and adaptive immune cells [39]. Similarly, the effect of T cells was not captured at this time point. It is known that preferential activation of T cell subtypes decreases myocarditis in otherwise susceptible mice [32,34,40]. Further characterization of subtypes of infiltrating immune cells and their related cytokines and chemokines may explain the higher immune cell composition in *Tnni3k* knockout mice and its later resolution.

This study focused on Tnni3k in CVB3 myocarditis, but in principle, Tnni3k may also influence cardiac immune response to infection by other viruses. It would also be relevant to address whether the inflammatory responses in non-viral heart diseases, such as autoimmune myocarditis or infarction, share the same involvement of Tnni3k.

Our mouse studies may have a direct homolog in human biology. We showed that K489R mice, in which there is normal expression of a kinase-dead single point mutation protein, have an elevated inflammatory response similar to full knockout mice (no protein expression). In the human population, the most common nonsynonymous *TNNI3K* variant is an isoleucine to threonine change at position 686, present at an allele frequency of approximately 2% worldwide and higher in specific subgroups [41]. Because of this prevalence, there are literally millions of people who are homozygous for this polymorphism, without any documented impact. Our biochemical studies show that the equivalent mutation introduced into the mouse protein results in severely compromised kinase activity [23]. While classified currently as a variant of unknown significance, it is possible that human carriers of the *TNNI3K* I686T polymorphism, or of others that have loss-of-function consequences, might be at higher risk of developing more severe myocarditis in response to viral infection.

Because TNNI3K is CM-specific and a kinase, the pharmaceutical industry has developed highly selective small-molecule TNNI3K kinase inhibitors, even in advance of having a known therapeutic purpose [42,43]. Because TNNI3K kinase activity suppresses viral myocarditis, an inhibitor of TNNI3K kinase activity would be expected to cause susceptibility to viral myocarditis, the same as the genetic knockouts used in this study. Given the endemic nature of viruses that can cause myocarditis, the combination of viral infection and TNNI3K inhibition could be problematic.

## Data Availability

The original contributions presented in this study are included in the article/Supplementary Materials. Further inquiries can be directed to the corresponding author.

## Supplementary Materials

The following supporting information can be downloaded at: https://www.mdpi.com/article/10.3390/jcdd13020069/s1, Figure S1: Immune cell infiltration is consistent through non-nearby sections of the heart; Figure S2: Tnni3k does not alter immune cell composition in uninfected mice.

## Abbreviations

The following abbreviations are used in this manuscript:

dpi	Days post-infection
CVB3	Coxsackievirus B3
