# Supplementary material for: Tnni3k Is Cardioprotective in Viral Myocarditis
Source: J Cardiovasc Dev Dis. 2026 Jan 30;13(2):69. doi: 10.3390/jcdd13020069 (PMC12941999; doi:10.3390/jcdd13020069)
Supplement: Supplementary file 1 [file jcdd-13-00069-s001.zip › jcdd-4095939-supplementary.pdf]

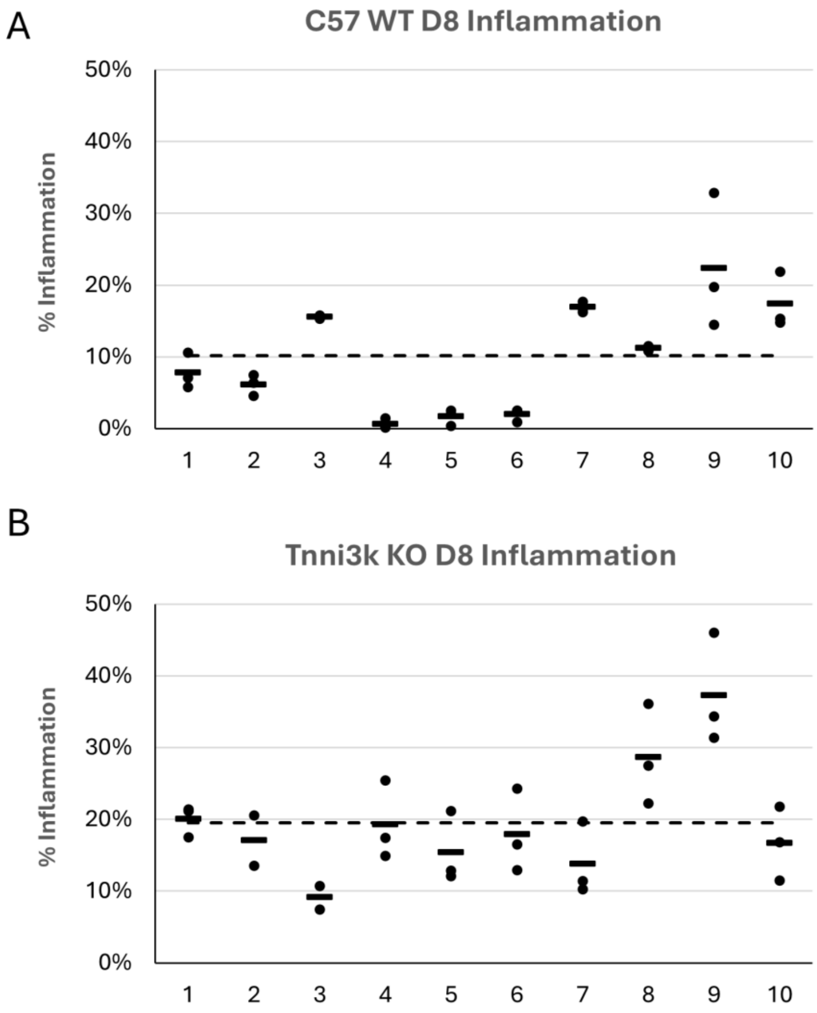

Supplementary Figure S1. Immune cell infiltration is consistent through non-nearby sections of the heart. Each of 2-3 individual sections per heart of 10 control (A) and 10 KO (B) mice was scored (dots) and the average of these (short dashes) was taken to represent the percent inflammation of that animal. The overall average for the groups of 10 mice (dotted line) is the same as in Figure 1C. Data only for 8 dpi are shown, but a similar degree of variability was seen for all samples at all time points shown in Figure 1C.

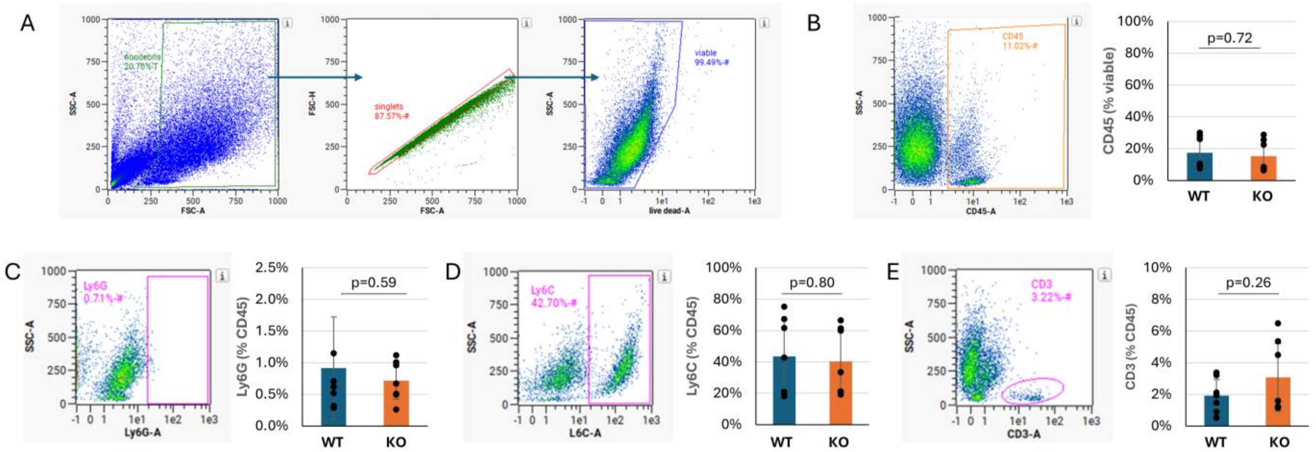

Supplementary Figure S2. Tnni3k does not alter immune cell composition in uninfected mice. **A.** Representative gating strategy for flow cytometry analyses. **B.** Density plot showing percentage of viable cells that are $CD45^+$, quantified to the right. **C-E.** Representative density plots showing percentages of $CD45^+$ cells that are $Ly6G^+$ (C), $Ly6C^+$ (D), and $CD3^+$ (E), with quantification to the right (n=7 for each genotype).
